# miR-125a-5p regulates the sialyltransferase ST3GAL1 in murine model of human intestinal campylobacteriosis

**DOI:** 10.1186/s13099-023-00577-6

**Published:** 2023-10-17

**Authors:** Angelina Kraski, Soraya Mousavi, Markus M. Heimesaat, Stefan Bereswill, Ralf Einspanier, Thomas Alter, Greta Gölz, Soroush Sharbati

**Affiliations:** 1 Institute of Veterinary Biochemistry, Freie Universität Berlin, Berlin, Germany; 2 Institute of Microbiology, Infectious Diseases and Immunology, Humboldt-Universität zu Berlin, and Berlin Institute of Health, Charité-Universitätsmedizin Berlin, Corporate Member of Freie Universität Berlin, Berlin, Germany; 3 Institute of Food Safety and Food Hygiene, Freie Universität Berlin, Berlin, Germany

**Keywords:** *Campylobacter jejuni*, *O*-glycosylation, microRNA, Mucin, Infection, Colonisation

## Abstract

**Background:**

Zoonotic microorganisms are increasingly impacting human health worldwide. Due to the development of the global population, humans and animals live in shared and progressively crowded ecosystems, which enhances the risk of zoonoses. Although *Campylobacter* species are among the most important bacterial zoonotic agents worldwide, the molecular mechanisms of many host and pathogen factors involved in colonisation and infection are poorly understood. *Campylobacter jejuni* colonises the crypts of the human colon and causes acute inflammatory processes. The mucus and associated proteins play a central host-protective role in this process. The aim of this study was to explore the regulation of specific glycosyltransferase genes relevant to differential mucin-type *O*-glycosylation that could influence host colonisation and infection by *C. jejuni*.

**Results:**

Since microRNAs are known to be important regulators of the mammalian host cell response to bacterial infections, we focussed on the role of miR-125a-5p in *C. jejuni* infection. Combining in vitro and in vivo approaches, we show that miR-125a-5p regulates the expression of the sialyltransferase ST3GAL1 in an infection-dependent manner. The protein ST3GAL1 shows markedly increased intestinal levels in infected mice, with enhanced distribution in the mucosal epithelial layer in contrast to naïve mice.

**Conclusion:**

From our previous studies and the data presented here, we conclude that miR-125a-5p and the previously reported miR-615-3p are involved in regulating the glycosylation patterns of relevant host cell response proteins during *C. jejuni* infection. The miRNA-dependent modulation of mucin-type *O*-glycosylation could be part of the mucosal immune response, but also a pathogen-driven modification that allows colonisation and infection of the mammalian host.

**Supplementary Information:**

The online version contains supplementary material available at 10.1186/s13099-023-00577-6.

## Background

The mammalian colon is covered by a constantly renewing, double mucus layer that forms the first line of defence in the colon. The main component of both layers is the highly glycosylated Mucin 2 (MUC2) which is produced, stored and secreted by goblet cells [1]. Increased mucus secretion in the crypts is a response to stress stimuli such as bacterial invasion [2, 3]. The elevated secretion facilitates the elimination of the pathogens by quickly flushing them away. Once secreted, MUC2 unfolds and builds a polymeric and viscous network due to numerously hydrated *O*-glycosylations connected to repeating domains rich in the amino acids proline, threonine, and serine (PTS-domains) [4]. The MUC2 glycosylation is a post-translational modification that takes place as a stepwise process in the Golgi apparatus [5]. Firstly, the Tn-antigen is created by *N*-acetylgalactosamine binding to serine or threonine of the protein backbone, then the galactosaminyltransferase C1GALT1 and *N*-acetylglucosaminyltransferases B3GNT6 and GCNT1-4 catalyse the elongation of the *O*-linked oligosaccharide chains [5, 6]. Various *O*-glycans can be bound by specific glycosyltransferases, forming long carbohydrate chains and different core structures with different functions. Mucin-type *O*-glycan chains can be expanded or branched by sialic acids on terminal, non-reducing ends expressed both on cell-surfaces and secreted glycoproteins. The transfer of sialic acid residues from a sugar nucleotide donor, mainly *N*-acetylneuraminic acid, to an acceptor substrate is catalysed by sialyltransferases such as ST3 beta-galactoside alpha-2,3-sialyltransferase 1 (ST3GAL1) and 2 (ST3GAL2) [7]. The negatively charged sialic acid improves water binding of MUC2 and prevents degradation of the protein backbone [8, 9].

Apart from host *O*-glycans, pathogenic bacteria also have characteristic oligosaccharides on their surface that serve host-pathogen interactions and are due to co-evolution with their hosts [2, 10]. Here, *O*-glycans on mucins can act as ligands for bacterial adhesins and specific connections between bacterial and MUC2 glycans can enhance adherence and invasion of intestinal pathogens. Some gastrointestinal pathogens, such as *Campylobacter jejuni*, are able to bind host mucins mediated by glycan-glycan interactions and thereby penetrate the otherwise sterile inner mucus layer invading and infecting the underlaying epithelium [2]. In humans, *C. jejuni* is the leading cause of foodborne bacterial gastroenteritis (campylobacteriosis) worldwide (http://www.who.int). In pigs and poultry though, the zoonotic bacteria can colonise the intestinal tract asymptomatically without causing disease [11]. Even though *C. jejuni* infections in humans are highly prevalent, only little is known about the pathomechanisms, and further research is needed to understand the molecular and regulatory backgrounds with special emphasis on host glycans and their modifications.

Previous work has already assessed that microRNAs (miRNAs) play an important role in the mammalian immune response after bacterial or viral infections [10, 12–14]. miRNAs are short, non-coding RNAs that are involved in regulating gene expression and silencing [15]. After binding their target sites, they initiate degradation or translational repression of the mRNA [5]. While one miRNA can mediate the activity of different genes, a single gene could also be regulated by several miRNAs, resulting in complex regulatory networks that control cellular processes such as differentiation, proliferation or immune response [15]. For instance, it has been shown that *Mycobacterium tuberculosis* induces the overexpression of miR-125b to block tumour necrosis factor (TNF) biosynthesis for a suppressed immune response [16]. Also, decreased expression of miR-125a-5p was associated with the presence of *Helicobacter pylori* in human gastritis [17]. Our recent study in a mouse model of human campylobacteriosis based on secondary abiotic IL10^−/−^ mice [18] has shown that the mammalian conserved miRNAs miR-615-3p and miR-125a-5p are dysregulated upon *C. jejuni* infection [10]. The study revealed that the identified miRNAs are mutually involved in regulation of several glycosyl- and sialyltransferases participating in mucin-type *O*-glycosylation in the murine colon. In the mentioned study, we mainly focused on the interaction between miR-615-3p and *St3gal2* and were able to demonstrate their molecular interaction in an infection-dependent context [19]. However, advanced knowledge about the role of these two miRNAs in gastrointestinal bacterial infections is still scarce.

Here, we investigated the *C. jejuni* infection dependent interaction between miR-125a-5p and the sialyltransferase ST3GAL1*,* a conserved target in both human and mouse. We identified target sites of miR-125a-5p in the 3’ untranslated region (UTR) of *St3gal1* and validated them in vitro. By applying the above-mentioned in vivo mouse model of human campylobacteriosis [18], we demonstrated that *St3gal1* and *B4galt1* possess anti-correlated gene expression compared to miR-125a-5p during *C. jejuni* infection. This was supported by increased ST3GAL1 protein levels in colon samples of infected mice.

## Results

### The sialyltransferase ST3GAL1 is a predicted target of miR-125a-5p

**Fig. 1:**
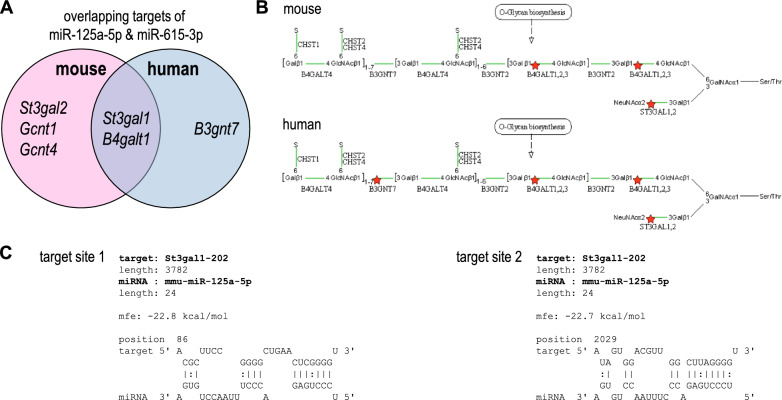
In silico predicted conserved murine and human targets of miR-125a-5p and miR-615-3p. **A** The Venn diagram presents interfaces of overlapping targets of miR-125a-5p and miR-615-3p within the mucin-type *O*-glycosylation pathway of which the glycosyltransferases *St3gal1* and *B4galt1* are conserved in human and mouse, respectively. **B** Excerpt of the KEGG signalling pathway enrichment analysis of mucin-type *O*-glycosylation in mouse and human [20]. Marked by the red asterisks are the specific steps in the glycosylation that might be modified by miR-125a-5p and miR-615-3p interaction. **C** Two binding sites of miR-125a-5p were identified in the 3’UTR of the murine *St3gal1* using RNAhybrid [21]

We have recently shown that the two miRNAs, miR-125a-5p and miR-615-3p, are dysregulated after intestinal *C. jejuni* infections [10]. For deciphering regulatory mechanisms of these miRNAs on mucin-type *O*-glycan biosynthesis, relevant human and murine target genes of the miRNAs were identified followed by KEGG pathway enrichment analysis [20] as described earlier [10, 12]. While only focussing on murine targets in the previous study, we have now expanded the enrichment analysis for both murine and human target genes. As shown in Fig. 1A, potential targets of both miRNAs within the mucin-type *O*-glycosylation pathway being conserved in both species were *St3gal1* and β1,4-galactosyltransferase *B4galt1*. However, *St3gal2, Gcnt4* and *Gcnt1* were only targeted by both miRNAs in mice, whereas the glucuronyltransferase *B3gnt1* was only a target in humans. In Additional file 1, all overlapping potential targets of miR-125a-5p and miR-615-3p in human and mouse are listed, including their pathways as determined by KEGG pathway enrichment analysis. As shown in Fig. 1B, the analysis pointed out specific sites of mucin-type *O*-glycan biosynthesis by the targeted transferases in mice and humans. While ST3GAL1 and 2 terminate the extension of the glycan chain by sialylation, B4GALT1 extends the core 2 structure by adding galactose residues. In Fig. 1B the intermediate steps in mucin-type *O*-glycosylation catalysed by ST3GAL1, ST3GAL2 and B4GALT1 were indicated (red asterisks), thereby forming potential interfaces with miR-125a-5p and miR-615-3p during *C. jejuni* infection. In previously published results, *St3gal2* was shown to be a target of miR-615-3p [10]. Furthermore, a clear anticorrelation between the expression of *St3gal1* and miR-125a-5p in the context of *C. jejuni* infection in vivo was observed*.* Therefore, we have mainly focused on this interaction in the analyses carried out here. Through RNAhybrid analysis [21], we have been able to identify two target sites of miR-125a-5p within the 3’ UTR of murine *St3gal1* (Fig. 1C). Both target sites possessed canonical interaction characteristics with minimal free energies ≤ − 22.7 kcal/mol.

### miR-125a-5p targets *St3gal1* in the murine intestinal cell line CMT 93

**Fig. 2:**
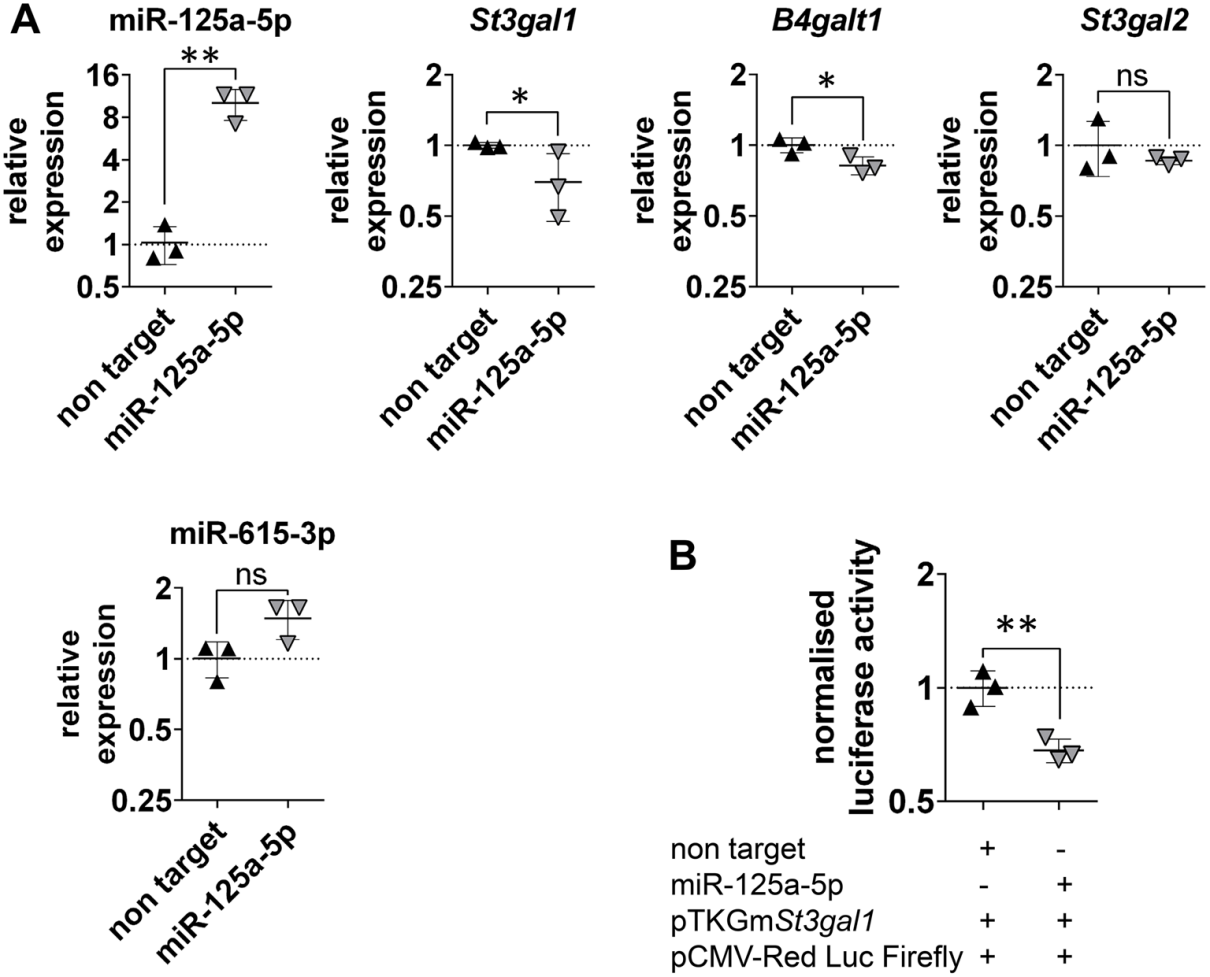
Specific interaction between miR-125a-5p and *St3gal1* was verified by RNAi and dual luciferase reporter assay. **A** Transfection efficiency of the murine intestinal cell line CMT 93 with miR-125a-5p mimics as well as expression of potential targets was evaluated by means of RT-qPCR. Most pronounced and significant decrease of the target *St3gal1* was detected after miR-125a-5p transfection. *B4galt1* showed significantly reduced levels. *St3gal2* showed no significant difference. miR-615-3p showed upregulated but not significant expression. Non-target miRNA was used as a control, fold changes were calculated relatively to the non-target control and normalised with HPRT and SDHA or SNORD44 and SNORD 47, respectively. Charts indicate means ± standard deviations (SD) of three biological samples with triple measurements. **B** Relative luciferase activity was determined in comparison to control non-target miRNA mimic. miR-125a-5p caused clear and significant activity of the reporter gene fused to the target sites compared with non-target transfected controls. Charts indicate means ± SD of three biological replicates with technical triplicates. Statistical significance is presented by asterisks compared to negative controls at each time point. *P ≤ 0.05, **P ≤ 0.01, unpaired t-test

We employed the murine intestinal cell line CMT 93 (ECACC 89111413) to examine the predicted interaction between miR-125a-5p and *St3gal1 *in vitro. The efficiency of miR-125a-5p transfection was determined by measuring its cellular levels by RT-qPCR. After transfection, CMT 93 showed approximately tenfold increased and significant (P ≤ 0.01) miR-125a-5p concentrations compared to non-target transfected controls (Fig. 2A). Since miRNAs are involved in gene silencing, anti-correlated expression of the target is anticipated after RNAi and indicates an interaction. We found that RNAi with miR-125a-5p mimics caused significantly decreased expression of *St3gal1* by 0.7-fold (P ≤ 0.05) in CMT 93 compared to non-target transfected controls (Fig. 2A). This confirmed our in silico analysis and pointed to *St3gal1* as a target of miR-125a-5p in mice.

Furthermore, we tested whether increased cellular miR-125a-5p levels had an effect on the transcripts of the potential targets *St3gal2* and *B4galt1* as well as the associated miR-615-3p. Although *B4galt1* was significantly downregulated 0.82-fold (P ≤ 0.05), no significant differences in *St3gal2* expression levels were observed between mimics and controls, though a trend towards decreased mRNA levels was found (Fig. 2A). Hence, these results point to *B4galt1* as a further target of miR-125a-5p. Interestingly, miR-615-3p also exhibited a trend towards increased expression (not significant) after miR-125a-5p transfection, suggesting synergistic effects of both miRNAs (Fig. 2A).

### Luciferase reporter assays prove specific interaction of miR-125a-5p with *St3gal1*

After screening the 3′ UTR of murine *St3gal1* for miR-125a-5p interaction sites by means of RNAhybrid [21], two binding sites were determined. The calculated minimal free energies for the interactions with miR-125a-5p were − 22.7 kcal/mol and − 22.8 kcal/mol, respectively (Fig. 1C). Dual reporter gene assays considering both identified *St3gal1* binding sites were performed as previously described [12]. After co-transfection of miR-125a-5p mimic together with the reporter plasmid (pTKGmSt3gal1) and the normalisation plasmid (pCMV-Red Firefly Luc), a significant 0.68-fold decrease (P < 0.01) in normalised luciferase activity (Luc_*Gaussia*_: Luc_*Red Firefly*_) was measured compared to non-target controls (Fig. 2B). The downregulated magnitude of the reporter gene fused to *St3gal1* binding sites was comparable to the reduction of *St3gal1* transcript levels in transfected CMT 93 cells by miR-125a-5p mimics. This experiment thus proved that the interaction between *St3gal1* binding sites and miR-125a-5p is specific.

### Murine *C. jejuni* infection model proves miR-125a-5p mediated *St3gal1* regulation

To investigate the physiological relevance of the above determined interaction between miR-125a-5p and the sialyltransferase *St3gal1 *in vivo, tissue samples of secondary abiotic IL10 ^−/−^ mice that had been infected with *C. jejuni* and naïve controls were compared.

**Fig. 3:**
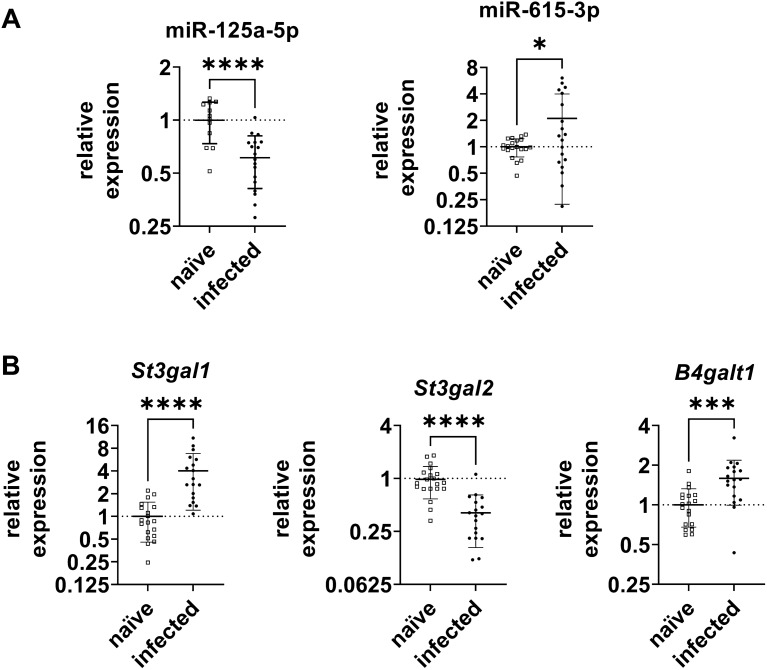
Relative gene expression of relevant miRNAs and their target mRNAs in secondary abiotic IL10 ^−/−^ mice colonic tissue sections six days post *C. jejuni* infection. 19 *C. jejuni* infected and 20 naïve control mice were included in the study. Each data point in the diagram represents an animal that showed confirmed expression in the respective analysis. **A** Upon *C. jejuni* infection, expression of miR-125a-5p was significantly decreased, whereas transcriptional levels of *St3gal1* and *B4galt1* were significantly enhanced. **B** miR-615-3p levels were significantly increased, while *St3gal2* was significantly downregulated. Expressions were relatively calculated to naïve controls and normalised with SNORD44 and SNORD47 or with HPRT and SDHA, respectively. For each individual, the mean was calculated based on triplicate measurements. Charts show mean ± SD of all individuals. Statistical significance is presented by asterisks compared to naïve controls. *P ≤ 0.05, ***P ≤ 0.001, ****P ≤ 0.0001, unpaired t-test

As shown in Fig. 3A, anticorrelated expression of miR-125a-5p and its target *St3gal1* was detected after *C. jejuni* infection. While miR-125a-5p was significantly downregulated by 0.6-fold (P ≤ 0.0001), a pronounced upregulation of *St3gal1* gene expression was observed after infection (> 4-fold; P ≤ 0.0001). Also, the expression of *B4galt1* was 1.6-fold upregulated (P ≤ 0.001) in the infected samples (Fig. 3A). Although the RNAi experiments performed above indicated that *St3gal2* is not a target of miR-125a-5p, we tested its expression in vivo. As shown in Fig. 3B, *St3gal2* was significantly downregulated by 0.4-fold (P ≤ 0.0001), while miR-615-3p was 2.1-fold increased (P ≤ 0.05) confirming our previously published data [10].

The marked and contrasting expression of *St3gal1* compared to miR-125a-5p confirmed our in vitro experiments and pointed out the specific role of the identified interaction in the context of intestinal *C. jejuni* infection in the applied murine model.

### The protein ST3GAL1 in the murine colon shows increased and corresponding levels after infection

**Fig. 4:**
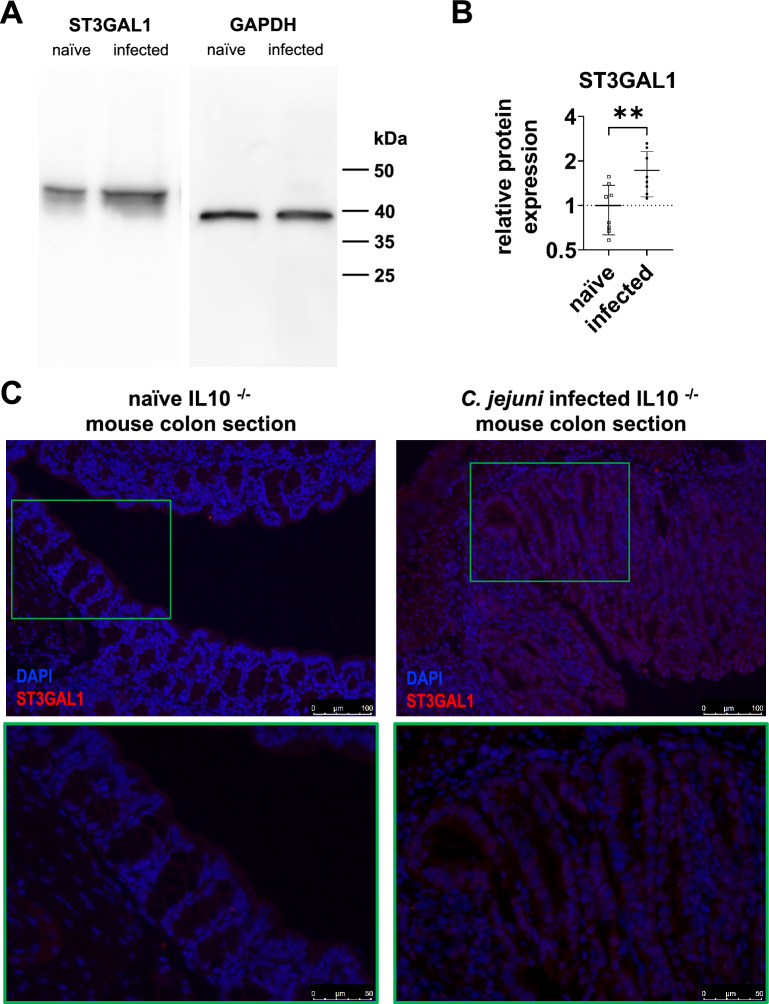
Increased colonic ST3GAL1 protein levels in secondary abiotic IL10 ^−/−^ mice six days post *C. jejuni* infection compared to naïve controls. **A** Increased ST3GAL1 protein levels were detected by western blots in pooled protein samples of infected mice. GAPDH is presented as the respective loading reference. **B** ST3GAL1 detection of eight *C. jejuni* infected and eight naïve mice colon samples were quantified by means of western blotting followed by densitometric analysis relative to the respective GAPDH signals as controls. Protein levels of ST3GAL1 in the infected group were significantly and 1.7-fold increased, compared to naïve controls. Charts show normalised mean ± SD in each group. Statistical significance is presented by asterisks. **P ≤ 0.01, unpaired t-test. **C** Localisation and enhanced ST3GAL1 expression in the colon of *C. jejuni* infected mice determined in representative immunofluorescent staining compared to naïve controls. ST3GAL1 was detected by immunofluorescent staining and shown in red using DyLight 594 whereas the nuclei were stained blue using DAPI. Enhanced red signal intensity and pronounced cytosolic distribution was detected in infected controls. The top row shows the overview, with the area outlined in green in the bottom row shown enlarged. Scale bars indicate 100 µm and 50 µm. Exposure time was identical for all colon sections and presented images are representative for three biological replicates tested

Following the interaction and gene expression analysis, the aim was to verify whether the infection-dependent downregulation of miR-125a-5p exerts an effect on the protein concentration and distribution of ST3GAL1 in colonic tissue. For this purpose, both western blots and immunofluorescence staining (IF) were performed with colonic tissue derived from infected and naïve mice, corresponding to the samples used for RT-qPCR analysis. As determined by western blots in pooled samples, ST3GAL1 protein levels were increased in infected tissue compared to respective naïve controls, while the levels of the reference protein GAPDH were similar in both groups (Fig. 4A). To statistically evaluate the magnitude of ST3GAL1 upregulation, individual western blots were performed from eight naïve and eight infected mice that were randomly selected and analysed using densitometry. The 1.7-fold significant increase in relative ST3GAL1 protein levels (P ≤ 0.01) in infected colonic tissue confirmed the transcriptional data (Fig. 4B). Western blot images of individual and pooled mice samples are shown in the Additional file 3.

Besides western blots, IF of ST3GAL1 in the corresponding colonic tissue sections was performed to assess protein abundance as well as localisation confirming the western blot data. An enhanced fluorescent signal of ST3GAL1 was detected in the *C. jejuni* infected group compared to naïve counterparts (Fig. 4C and Additional file 3), with an abundant cytosolic detection of ST3GAL1 in the enterocytes lining the entire length of the crypts of infected sections. In contrast, ST3GAL1 was more abundant in the upper part of the crypts towards the intestinal lumen in the control tissue. Additionally, ST3GAL1 was mainly localised on the apical side of the cell membrane in the naïve samples, whereas the fluorescence signal in the infected samples was more intense and evenly distributed over the entire cytosol of the cell (Fig. 4C). Images of individual sections from both groups and negative controls of the tissue sections, to ensure the specificity of the detected ST3GAL1 signal, are shown in the Additional file 5.

## Discussion

Some pathogenic bacteria such as the zoonotic *C. jejuni* can infiltrate and penetrate the protective mucus layer in the colon and infect the mucosa. Even though *C. jejuni* is such a relevant human pathogen, the mechanisms underlying the infection have not been entirely unravelled yet and questions regarding colonisation and infection as well as the role of the host intestinal immune response remain unanswered to date. One major aspect within these processes forms the mucus. However, the role of the highly glycosylated MUC2, the main component of both colonic mucus layers, is not fully understood. In this study, we aimed to gain a more detailed insight into the regulatory mechanisms of mucin-type *O*-glycosylation in *C. jejuni* infections using a mouse model of human campylobacteriosis.

The importance of well-functioning MUC2 layers as a first line of defence against pathogens in the colon was revealed in previous studies [22–24]. As a response to *C. jejuni* infection, we have shown here that enzymes involved in the mucin-type *O*-glycosylation exhibit altered intestinal expression, which may lead to differential MUC2 glycosylation and consequently affect the protective properties of the glycoprotein. This was also reported upon infections with other pathogenic species. For example, *H. pylori* can modulate *O*-glycosylation, leading to changes in mucosal glycans and enhanced mucin sialylation due to dysregulations of sialyltransferases [25–28]. In the first line of *H. pylori* infection, the adhesion of the pathogen is mediated by antigen binding adhesin (BabA) binding at H-type 1 and Lewis b structures present on mucin (28]. The increase of sialylated mucin structures during the infection process promotes a closer membrane attachment of *H. pylori* mediated by the interaction of the sialic acid binding adhesin (SabA) with sialylated mucin structures, facilitating the infection [28]. This might also be the mechanism how *C. jejuni* penetrates the inner mucus layer and enables infection of the epithelium.

In the colon, ST3GAL1 primarily adds sialic acid to the core 1 *O*-glycans, a dominant core structure in mucin glycosylation, while ST3GAL2 transfers sialic acid to the terminal galactose residues found in glycoconjugates. B4GALT1, in contrast, catalyses the transfer of galactose to non-reducing ends of the sugar chain [29–31]. *C. jejuni* dependent dysregulation of miR-125a-5p and the previously reported miR-615-3p as well as the targeted glycosyltransferases in the applied mouse model suggest that a miRNA-dependent modification of mucin-type *O*-glycosylation takes place in the colon upon infection. It is still to be determined whether this is caused by *C. jejuni* directly or triggered by host immune responses. On the one hand, it was shown that modification of mucins and the increased mucus secretion is controlled by the epithelial cells responding to signals from the innate and adaptive immune system [26]. Pathogens have developed numerous mechanisms to penetrate the sterile inner mucus layer, thus, an altered mucin structure could serve as a host response preventing infection. This was seen in *H. pylori* infections and could also be assumed in infections with *C. jejuni* [32]. However, the altered *O*-glycosylation could also be pathogen-derived and may result in diminished mucin barrier function facilitating the infection. These alterations could affect colonisation and infection by the pathogen.

In humans, *Campylobacter* causes symptoms such as severe diarrhoea and abdominal cramping [11]. During infection, *Campylobacter* trigger a range of immune responses and studies have shown that miRNAs are involved in regulating immune responses in several ways. The mechanisms known so far range from regulation of inflammation-dependent genes to control of apoptosis of infected host cells [10, 13, 33]. Understanding miRNA function can provide new insights into the pathogenesis of microorganisms. Here, the role of miR-125a-5p in infections with bacterial pathogens is of particular interest. In *Mycobacterium avium* infections, miR-125a-5p was overexpressed in human macrophages as part of the innate immune response to the bacterial infection [34]. On the other hand, upregulated miR-125a in *Mycobacterium tuberculosis* infections was associated with enhanced bacterial survival in human and murine macrophages [35]. Furthermore, *H. pylori* infections in humans were accompanied by decreased miR-125a-5p expression [17] and downregulation of miR-125a in mesenteric lymph nodes of piglets after *Salmonella* Typhimurium infection was identified, triggering diverse immune responses [36]. Finally, *C. jejuni* infections in mice have been recently found to be associated with reduced miR-125a-5p and enhanced miR-613-3p expression in the colon [10]. Interestingly, all studies that have focussed on infections with intestinal pathogens revealed decreased expression of miR-125a-5p. In the present study, we focused on the interaction of miRNA-125a-5p with putative targets conserved in human and mice to emphasise its relevance in *C. jejuni* infections. In silico analysis determined two overlapping targets of both dysregulated miRNAs involved in mucin-type *O-*glycosylation being conserved in human and mice, *St3gal1* and *B4galt1*. Here, miR-125a-5p was selected because of its known involvement in several bacterial infections, including *C. jejuni* infections [10, 17].

Decreased gene expression of *St3gal1* and *B4galt1* was observed after RNAi with miR-125a-5p mimics in our in vitro transfection experiments. Similar counter-regulated expression profiles of miR-125a-5p with *St3gal1* and *B4galt1* mRNAs were also observed in colonic tissue of the infected mice. Therefore, it is likely that expressions of both genes are negatively regulated by miR-125a-5p. In contrast, expression of *St3gal2* was not significantly regulated after RNAi with miR-125a-5p mimics and therefore does not seem to be a target of miR-125a-5p. The fact that miR-615-3p tends to be upregulated after miR-125a-5p transfection suggests a synergistic mode of action of both miRNAs. However, the underlying regulatory mechanisms still need to be investigated. Overall, miRNAs can play an important role in regulating the expression of glycosyltransferases during infection, and understanding these interactions can not only provide new insights into the pathogenesis of infectious diseases but also help to develop novel therapeutics.

Apart from matching mRNA and protein levels of ST3GAL1 after intestinal infection of mice, we determined the localisation of ST3GAL1 in the infected tissue and furthermore, at the cellular level. Mucin-type *O*-glycosylation takes place in the Golgi apparatus, and sialylated glycoproteins are stored in granules before being exported to the cell membrane or secreted [1, 37]. IF staining showed cytoplasmic ST3GAL1 signal, suggesting concordant localisation of ST3GAL1 in the Golgi apparatus. As adhesins of various pathogens can bind to sialic acids on mucins, abundance of ST3GAL1 could lead to increased integration of sialic acids in MUC2 glycans enhancing pathogen binding [26, 38]. The observation that the glycosyltransferases are dysregulated after interacting with the miRNAs during infection with *C. jejuni* supports our hypothesis that a miRNA-dependent modification of the *O*-glycosylation of MUC2 or other signalling proteins might take place here. Further research will focus on altered glycosylation pattern of mucin after *C. jejuni* infection.

Conventional laboratory mice with a complex commensal gut microbiota are not susceptible to *C. jejuni* infection, whereas secondary abiotic IL10 ^−/−^ mice generated upon antibiotic pre-treatment can not only be infected by the enteropathogens, but also display key features of human campylobacteriosis including acute enterocolitis within 6 days post-infection [18]. Although the model simulates the course of the disease in humans well, there are limitations, as in any translational model. Thus, the results cannot be completely transferred to human conditions and differences between mice and humans need to be considered. There are clear species differences in the extent of intestinal colonisation by *C. jejuni*. In contrast to humans and secondary abiotic mice, the enteropathogens colonise the intestinal tract of poultry and pigs asymptomatically without causing clear clinical signs of infection. Here, comparative studies of the interactions investigated may reveal possible differences between the animal species and humans to understand why the aforementioned animals are less susceptible to *C. jejuni*. This would even have applied effects on food safety, because a better understanding of the factors that contribute to colonisation can be used to reduce the load of zoonotic agents in the food chain.

The two miRNAs studied here and previously, miR-125a-5p and miR-615-3p, could be involved in the colonisation and infection process of the gut by *C. jejuni*. Through their targets, they could be part of the mucosal and cellular host responses upon bacterial infection. Modified mucin-type *O*-glycosylation could be a direct mucosal response to hinder pathogenic colonisation and infection. On the contrary, *C. jejuni* could actively induce dysregulation of miRNAs facilitating host cell adhesion and invasion.

## Conclusion

The aim of our study was to elucidate the regulatory effects of miR-125a-5p during *C. jejuni* infection. We determined mutually conserved targets of miR-125a-5p and miR-615-3p in humans and mice, namely the sialyltransferase *St3gal1* and the glycosyltransferase *B4galt1*, both known to be involved in mucin-type *O*-glycosylation. Here, we focussed on *St3gal1* and confirmed it as a bona fide target of miR-125a-5p. Moreover, our in vivo analysis showed that miR-125a-5p as well as *St3gal1* are dysregulated upon *C. jejuni* infection in susceptible mice. The interactions studied here may not only be involved in the modification of mucus glycosylation but could also target cellular signalling proteins. This study therefore provides a basis for better understanding molecular factors that are important for colonisation and infection of *C. jejuni*.

## Methods

### Animal experiment

In vivo experiments were carried out by the Forschungseinrichtungen für Experimentelle Medizin (FEM, Charité-Universitätsmedizin Berlin) and according to the European Guidelines for animal welfare (2010/63/EU) following agreement by the commission for animal experiments headed by the “Landesamt für Gesundheit und Soziales” (LaGeSo, Berlin, registration number G0104/19). Animal welfare was monitored twice a day. For the experiment, 39 secondary abiotic IL10^−/−^ mice (C57BL/B10 background) were divided into two groups, *C. jejuni* infected and naïve, matched by age and sex [18]. In total, 19 animals were infected with 10^9^ colony forming units (CFU) of *C. jejuni* strain 81–176 in 0.3 ml sterile phosphate-buffered saline (PBS, Thermo Fisher Scientific, Waltham, MA, USA) on days 0 and 1 by oral gavage while 20 animals were not infected as controls. The mice were sacrificed six days post infection and ex vivo biopsies of the colon were collected under aseptic conditions. For the gene expression and protein analysis, the colon tissue samples were stored in Allprotect Tissue Reagent (Qiagen, Venlo, Netherlands) or liquid nitrogen, respectively and stored at − 80 °C. For the histopathological analysis, the biopsies were immediately fixed in 5% formalin and embedded in paraffin.

### Cell line and culture conditions

The murine rectal carcinoma cell line CMT 93 (ECACC 89111413) was cultured in a humidified atmosphere at 37 °C and 5% CO_2_ in Dulbeccco’s Modified Eagle’s Medium (DMEM) with 4.5 g/l Glucose and l-Glutamine (Gibco, Grand Island, NY, USA). The medium was supplemented with 100 µg/ml Gentamicin and 10% (v/v) fetal calf serum superior (Sigma-Aldrich, Darmstadt, Germany). The cells were grown in 75 cm^2^ tissue culture flasks (Sarstedt, Nümbrecht, Germany) until approximately 80% confluence was reached.

### RNA-isolation and RT-qPCR

Total RNA was isolated from the murine colonic tissue stored in Allprotect and CMT 93 cells using the miRVana Isolation Kit (Life Technologies, Carlsbad, CA, USA). Then, cDNA was synthesised from individual tissue samples or cell lysates. For this, the isolated total RNA was firstly treated with RNase-free DNase I (NEB GmbH, Frankfurt a/M, Germany) to remove residual genomic DNA. Then, 1 µg total RNA was applied for reverse transcription using 200 U M-MuLV Reverse Transcriptase (Thermo Fisher Scientific), 0.2 μg random hexamer primer (Thermo Fisher Scientific), 200 μM of each deoxynucleotide triphosphate (dNTP) (Thermo Fisher Scientific) and 1 × supplied RT buffer (Thermo Fisher Scientific). To verify the absence of genomic DNA, control samples were treated as just mentioned but without M-MuLV Reverse Transcriptase. Then, RT-qPCRs were carried out. The qPCR of the mRNAs was performed with the Blue S’Green qPCR Kit (Biozym Scientific GmbH, Hessisch Oldendorf, Germany) under following cycling conditions: amplification was conducted at 95 °C for 2 min, followed by 40 cycles with 15 s at 95 °C, 20 s at 60 °C and 30 s at 70 °C. For quality control, subsequent melting curve analysis was assessed by 95 °C for 15 s, 65 °C for 1 min, ramping from 65 to 95 °C at 5 °C/min. For the miRNA qPCR, the SensiFAST SYBR® Hi-ROX Kit (Bioline, Meridian Bioscience International Limited, Cincinnati, OH, USA) was used and cycling conditions comprised real-time analysis at 95 °C for 2 min, followed by 40 cycles with 15 s at 95 °C, 20 s at 60 °C and 30 s at 72 °C. Subsequent melting curve analysis was assessed by 95 °C for 15 s, 60 °C for 1 min, ramping from 60 to 95 °C at 5 °C/min. For RT-qPCR of the miRNAs, the miR-Q method [39] was used with specific RT-6-primer and corresponding reverse PCR primer (Additional file 2). All relative gene expression was calculated using the ∆∆Ct method [40] as described before [10]. Stability of the reference was tested using geNorm [41]. The HPRT and SDHA genes served as references for mRNA expression and the small RNAs SNORD44 and SNORD47 for miRNA expression. The mean Ct values of the target as well as the geometric mean of all Ct-values of the two respective references were calculated for each sample from three technical replicates. The ∆Ct is equal to the difference between the Ct of the target and the geometric mean of both references. The ∆∆Ct were calculated relative to controls.

All oligonucleotides in this study were synthesised by Sigma-Aldrich. Sequences, amplification efficiencies and primer concentrations and can be found in Additional file 2.

### Western blot

Colonic tissue samples derived from the animal experiment were lysed in cold RIPA buffer (Cell Signaling Technology (CST), Danvers, MA, USA), supplemented with protease inhibitor cocktail (CST) and protein was isolated. Protein concentration was quantified by using the 2D Quant Kit (Qiagen). 30 µg of protein was loaded onto a 12% sodium dodecyl sulfate-polyacrylamide (SDS) gel for electrophoresis. Then, they were transferred onto a polyvinylidene fluoride membrane (PVDF) (GE Healthcare, Buckinghamshire, UK) by means of semi-dry blotting. To prevent unspecific binding of the antibody, the membrane was blocked in 5% (w/v) bovine serum albumin (Sigma-Aldrich) in TBST (Tris–HCl-buffer with 0.1% (v/v) Tween-20) for 1.5 h at room temperature. The membrane was incubated with the primary antibody Rabbit anti-ST3GAL1 (Novus Biologicals, bio-techne, Minneapolis, MN, USA, NBP1-62540) 1:500 or Rabbit anti-GAPDH (CST, #5174) 1:2000 in 5% BSA in TBST at 4 °C overnight. After three washes in TBST for 15 min each, the membrane was probed with the secondary horseradish peroxidase (HRP)-linked anti-rabbit IgG antibody (1:2500 in 5% BSA in TBST, CST, #7074) for 2 h at room temperature. After three additional washes with TBST (Tris–HCl-buffer with 0.1% (v/v) Tween-20) for 15 min, immuno-reactive proteins were detected with the Amersham™ ECL Select™ Western Blotting Detection Reagent (GE Healthcare). Exposure conditions were identical for every blot. Protein quantification was performed by densitometry using the software BIO-1D (Vilber, Collégien, France). Here, a defined area was selected to be used on all lanes in each blot for 8 *C. jejuni* infected and 8 naïve mice colon samples and densitometry was measured. The optical density was determined by the software and the background was subtracted. The pixel density values of the ST3GAL1 signals were then normalised to the respective GAPDH signals and the fold change calculated on the mean of the controls. The raw pixel density values can be found in the Additional file 4.

### Histopathology and immunofluorescence

Murine colon tissue sections from the animal experiment were immediately fixed in 5% formalin and embedded in paraffin. 5 µm serial sections were cut using the Epredia™ HM 340E Electronic Rotary Microtome (Thermo Fisher Scientific). The paraffin was melted at 60 °C for 45 min and the tissue sections deparaffinated in Roticlear (Carl Roth, Karlsruhe, Germany). Rehydration of the tissue was reached through a graded series of ethanol followed by rinsing with distilled water and PBS (pH 7.4, Sigma-Aldrich). For IF, antigen retrieval was carried out by incubating the sections in boiling citrate buffer for 15 min followed by 20 min of cooling down. Non-specific binding was blocked with 1% (v/v) BSA (Sigma-Aldrich) in PBST (0.1% (v/v) Tween-20 in PBS) for 1 h at room temperature. Then, the sections were incubated with a 1:50 dilution of the Rabbit anti-ST3GAL1 primary antibody (Novus Biologicals, NBP1-62540), in 1% (v/v) BSA in PBST overnight at 4 °C. Negative controls were treated with 1% (v/v) BSA in PBST without the primary antibody. After three washes with PBS for 10 min, the primary antibody was detected with goat anti-rabbit IgG DyLight 594 secondary antibody (1:400, diluted in 1% (v/v) BSA in PBST, Thermo Fisher Scientific, #35561) for 1 h at room temperature. Following three washes with PBS, the nuclei were counterstained with 200 ng/ml 4′, 6-diamidin-2-phenylindol (DAPI, Sigma-Aldrich) in PBS for 5 min at room temperature. Subsequently, slides were washed in PBS and mounted with Prolong™ Diamond Anti-fade Mountant (Life Technology). For IF, the Leica DMI6000B inverted microscope with the compatible Leica LAS-X software (Leica, Wetzlar, Germany) was used. All images were taken under identical microscope- and camera-settings. Experiments were carried out with three randomly selected biological replicates per group. Images were taken at least from three random areas per section and more than ten images per area were selected. The background was subtracted in each image.

### RNAi and luciferase reporter assay

For both assays, CMT 93 cells were cultured as mentioned above. RNAi transfections of three biological replicates were performed using electroporation with the Nucleofector Technology (Lonza AG, Köln, Germany) as previously described with few modifications [41]. 1 × 10^6^ cells were mixed with 50 pmol of miR-125a-5p miRNA mimic (Qiagen, MSY0000443) or 50 pmol non-target siRNA (Dharmacon Lafayette, CO, USA, D-001810-03-05) as a control. After 24 h, the cells were washed with PBS and lysed for RNA isolation.

The dual luciferase reporter assays were carried out as previously mentioned [10, 41]. For the generation of the reporter plasmids, the in silico identified target sites of murine *St3gal1* were assembled using the hybridised oligonucleotides NotI-mmuSt3gal1ts-sense and XbaI-mmuSt3gal1ts-antisense from Sigma-Aldrich (Additional file 2). These target sites were cloned in pTK-Gluc (NEB GmbH) plasmid using the appropriate restriction enzymes *Not*I and *Xba*I (both NEB GmbH). For endotoxin-free reporter plasmids of the resulting derivative pTKGmSt3gal1, NucleoBond Xtra Midi Plus EF (Macherey–Nagel GmbH & Co. KG, Düren, Germany) was used. For the luciferase assay, 3.8 × 10^5^ CMT 93 cells were inversely transfected with 3.25 µg of endotoxin-free pTKGmSt3gal1 and for normalisation, 350 ng pCMV-Red Firefly Luc (Thermo Fisher Scientific, 16156) together with 200 pmol miR-125a-5p miRNA mimic (Qiagen) on a 6-Well Plate (Sarstedt) using 7.5 µl Lipofectamin 3000 (Thermo Fisher Scientific) and 2.5 µl P3000 Reagent (Thermo Fisher Scientific). The transfected cells were cultured for 48 h. As a negative control, 200 pmol non-target siRNA (Dharmacon Lafayette) was transfected. Gaussia-Firefly Luciferase activity was detected in three biological and three technical replicates with the Pierce Gaussia-Firefly Luciferase Dual Assay Kit (Thermo Fisher Scientific). For the dual luciferase reporter assays, the CLARIOstarPlus plate reader (BMG Labtech, Ortenberg, Germany) was used under following conditions: top optic, number of multichromatics: 2, number of kinetic widows: 1, number of cycles: 1, measurement interval time: 1.27, emission Luciferase-Red Firefly: 623 nm, emission Luciferase-Gaussia: 485 nm.

### In silico and statistical analysis

Mutual targets of miR-125a-5p and miR-615-3p in human and mouse were identified as well as data and pathway analysis performed as described earlier [10, 12]. Briefly, overlapping targets of both miRNAs were determined for mouse and human by miRmap followed by KEGG based pathway enrichment analysis. Conserved mutual targets involved in ‘Glycosaminoglycan biosynthesis—keratan sulfate’ (hsa00533 and mmu00533) and mucin type *O*-glycan biosynthesis (mmu00512) were determined. RNAhybrid was used to predict target sites of miRNA [21]. Normal distribution of data was assessed by the Anderson–Darling test. The unpaired t-test was used in this study to compare each treatment with control. All tests were conducted applying GraphPad Prism version 8.00 (GraphPad Software, La Jolla California USA, www.graphpad.com). Asterisks in figures summarize P values (*P ≤ 0.05; **P ≤ 0.01; ***P ≤ 0.001; ****P ≤ 0.0001).

### Supplementary Information

**Additional file 1. **Raw data of the in silico analysis using KEGG pathway enrichment analysis. Sheet 1 shows overlapping targets of miR-125a-5p and miR-615-3p as well as their pathways in humans. Sheet 2 shows overlapping targets of miR-125a-5p and miR-615-3p and their pathways in mice. Overlapping targets conserved in both species are highlighted in bold.

**Additional file 2. **All oligonucleotides used in this study. Shown are the primer sequences, amplification efficiencies, primer concentrations and the determined region of ST3GAL1 where miR-125a-5p possibly binds cloned in the plasmid used for Luciferase-Assay.

**Additional file 3. **Randomly selected tissue samples in pooled and individual blots. (a,b) Western blot detection of ST3GAL1 in eight naïve (n1-8) and eight *C. jejuni* infected (i1-8) secondary abiotic IL-10^−/−^ mouse colon sections. GAPDH is shown as the respective loading reference in the same samples. (c) Western blot detection of ST3GAL1 of pooled protein samples of four naïve (n1-4 and n5-8) and four *C. jejuni* infected (i1-4 and i5-8) secondary abiotic IL-10^−/−^ mouse colon sections and pooled protein samples of all eight naïve (n1-8) and eight infected (i1-8) sections. GAPDH is shown as the respective loading reference in the same pooled samples.

**Additional file 4. **Pixel density values of individual and pooled western blots as well as the selected area used for the determination of ST3GAL1 expression in colonic tissue samples of naïve and *C. jejuni* infected mice. Sheet 1 shows the pixel density values of the first four individual naïve (n1-4) and *C. jejuni* infected (i1-4) samples, sheet 2 of the second four individual naïve (n5-8) and *C. jejuni* infected (i5-8) samples. In sheet 3 values of all pooled samples (n1-4, i1-4, n5-8, i5-8, n1-8 and i1-8) are shown.

**Additional file 5. **Detection of immunofluorescently stained ST3GAL1 in secondary abiotic IL-10^−/−^mice upon *C. jejuni* 81–176 infection. ST3GAL1 is shown in red and nuclei were stained blue using DAPI. (a + b) Immunofluorescent staining of ST3GAL1 in two individual experiments of naïve mouse colon sections. (c + d) Two individual *C. jejuni* infected mouse colon sections immunostained with ST3GAL1. (e) Negative controls of naïve colonic tissue sections. (f) Negative controls of colon tissue sections infected with *C. jejuni*. Scale bars indicate 100 µm and 50 µm (magnification).

## Data Availability

All data generated or analysed during this study are included in this published article and its supplementary information files.
