# Supplementary figures and images for: miR-125a-5p regulates the sialyltransferase ST3GAL1 in murine model of human intestinal campylobacteriosis
Source: Gut Pathog. 2023 Oct 17;15:48. doi: 10.1186/s13099-023-00577-6 (PMC10583435; doi:10.1186/s13099-023-00577-6)

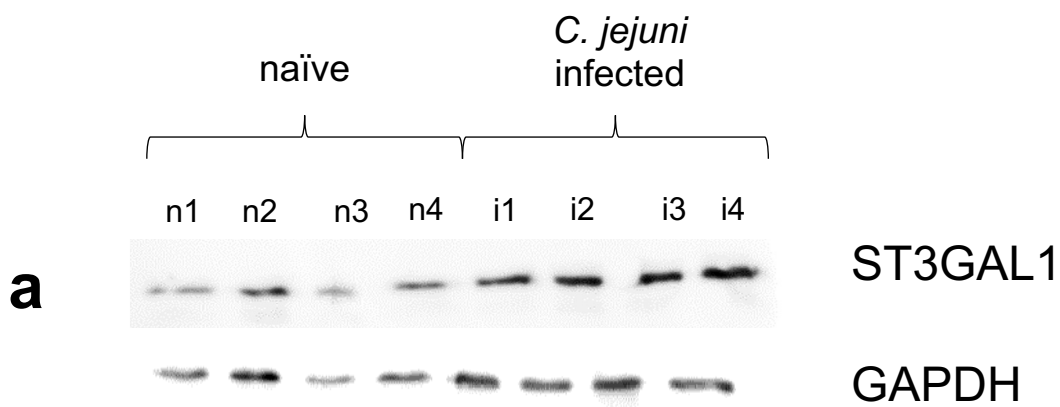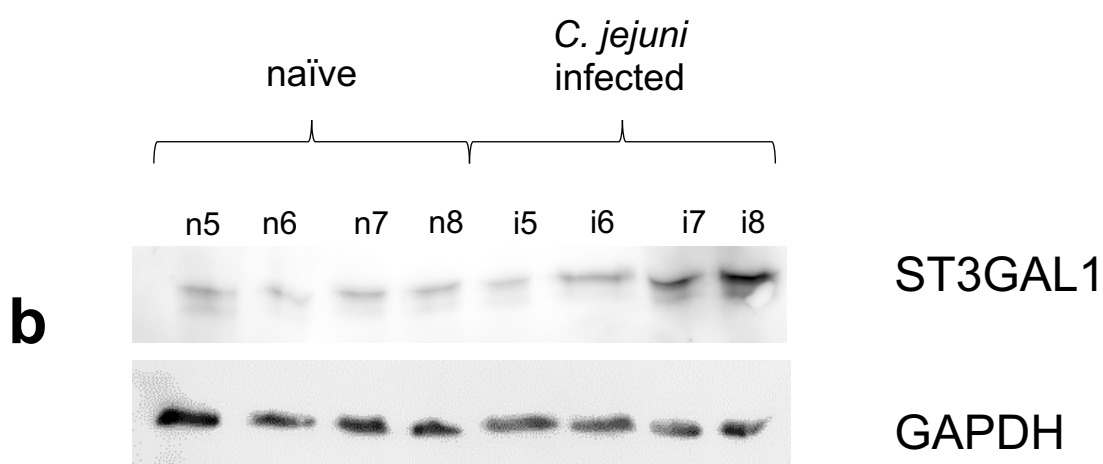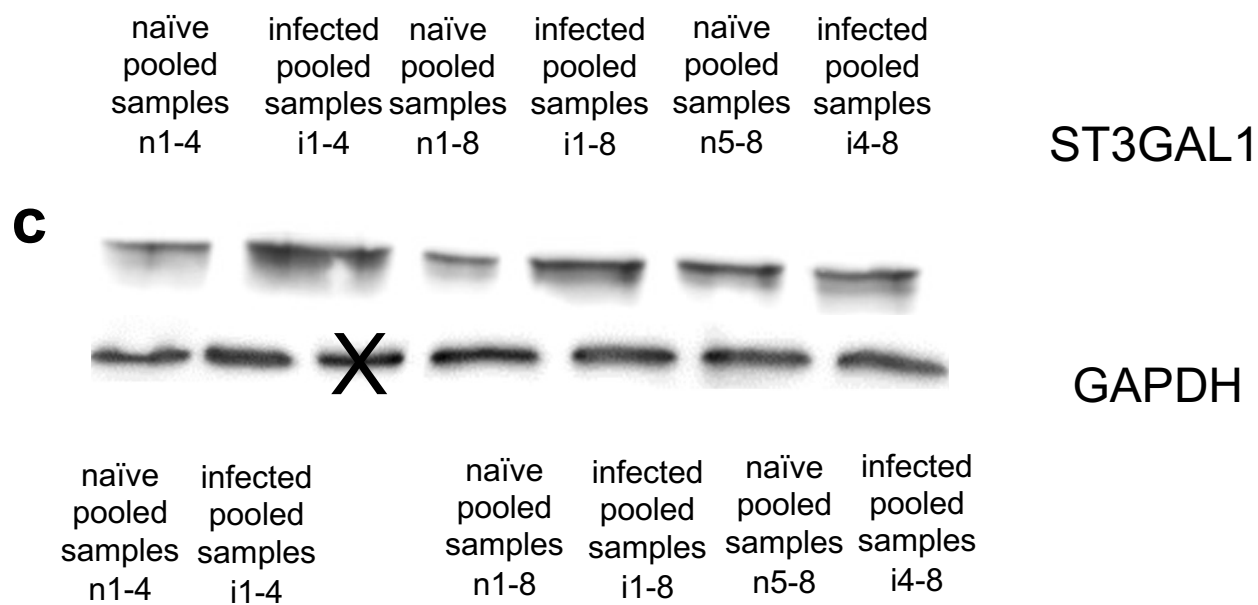

Supplement: Supplementary file 3 — Additional file 3. Randomly selected tissue samples in pooled and individual blots. (a,b) Western blot detection of ST3GAL1 in eight naïve (n1-8) and eight C. jejuni infected (i1-8) secondary abiotic IL-10−/− mouse colon sections. GAPDH is shown as the respective loading reference in the same samples. (c) Western blot detection of ST3GAL1 of pooled protein samples of four naïve (n1-4 and n5-8) and four C. jejuni infected (i1-4 and i5-8) secondary abiotic IL-10−/− mouse colon sections and pooled protein samples of all eight naïve (n1-8) and eight infected (i1-8) sections. GAPDH is shown as the respective loading reference in the same pooled samples. [file 13099_2023_577_MOESM3_ESM.pdf]
