# Supplementary material for: miR-125a-5p regulates the sialyltransferase ST3GAL1 in murine model of human intestinal campylobacteriosis
Source: Gut Pathog. 2023 Oct 17;15:48. doi: 10.1186/s13099-023-00577-6 (PMC10583435; doi:10.1186/s13099-023-00577-6)

naïve IL10 $^{-/-}$
mouse colon section

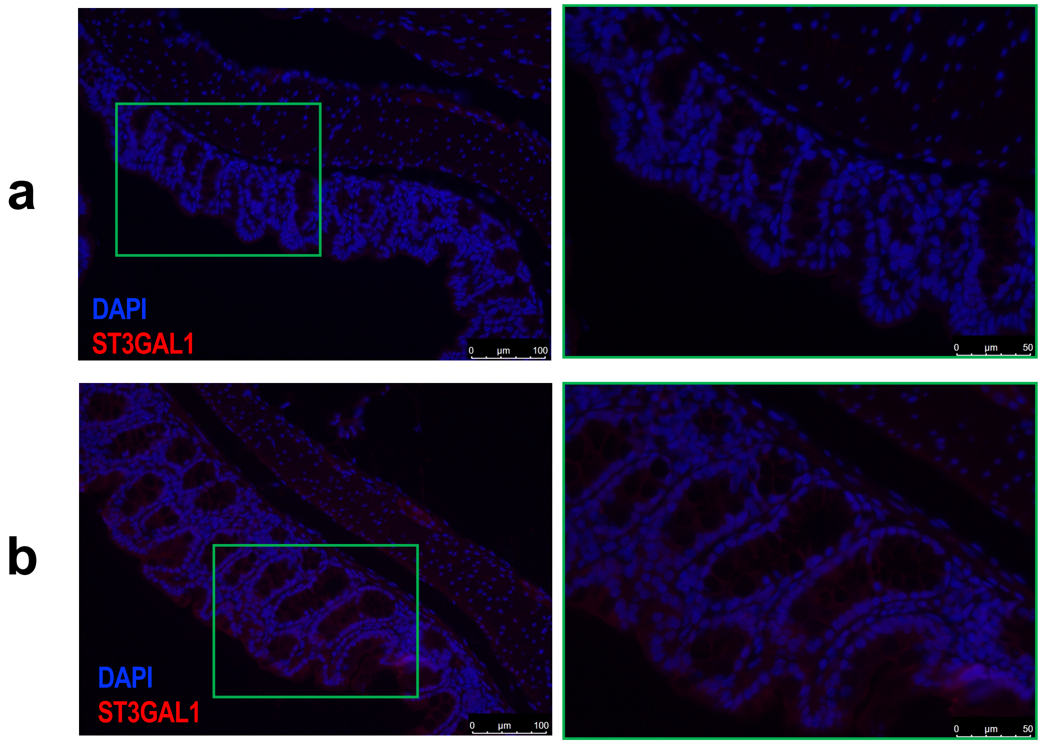

C. jejuni infected IL10 $^{-/-}$
mouse colon section

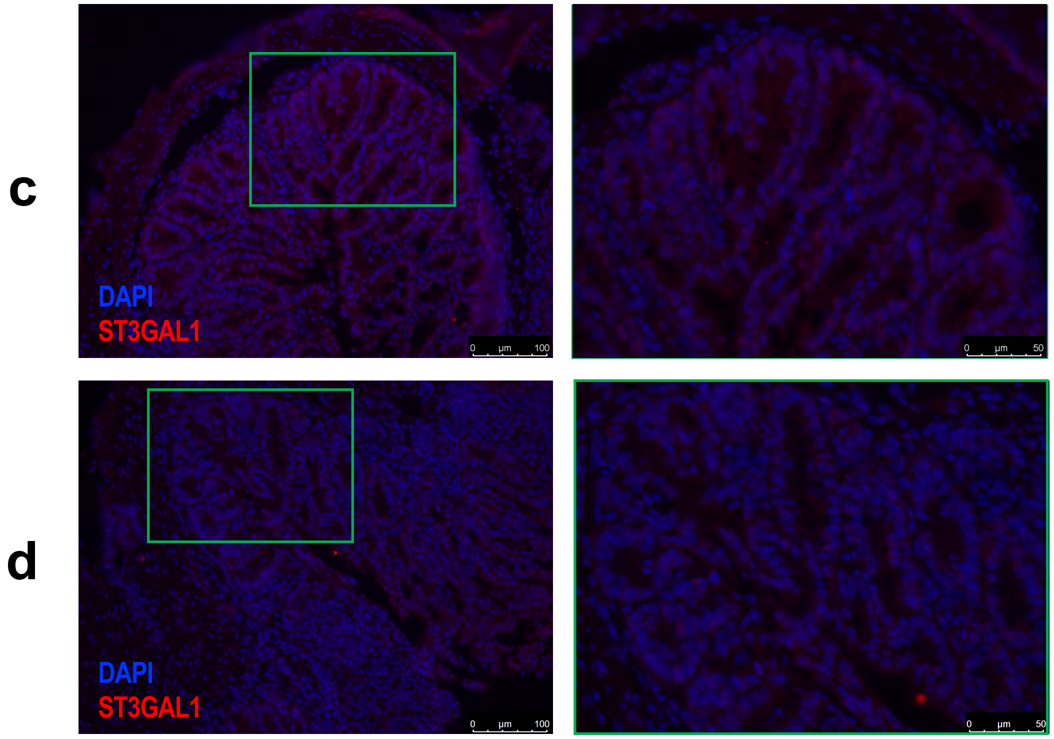

negative controls

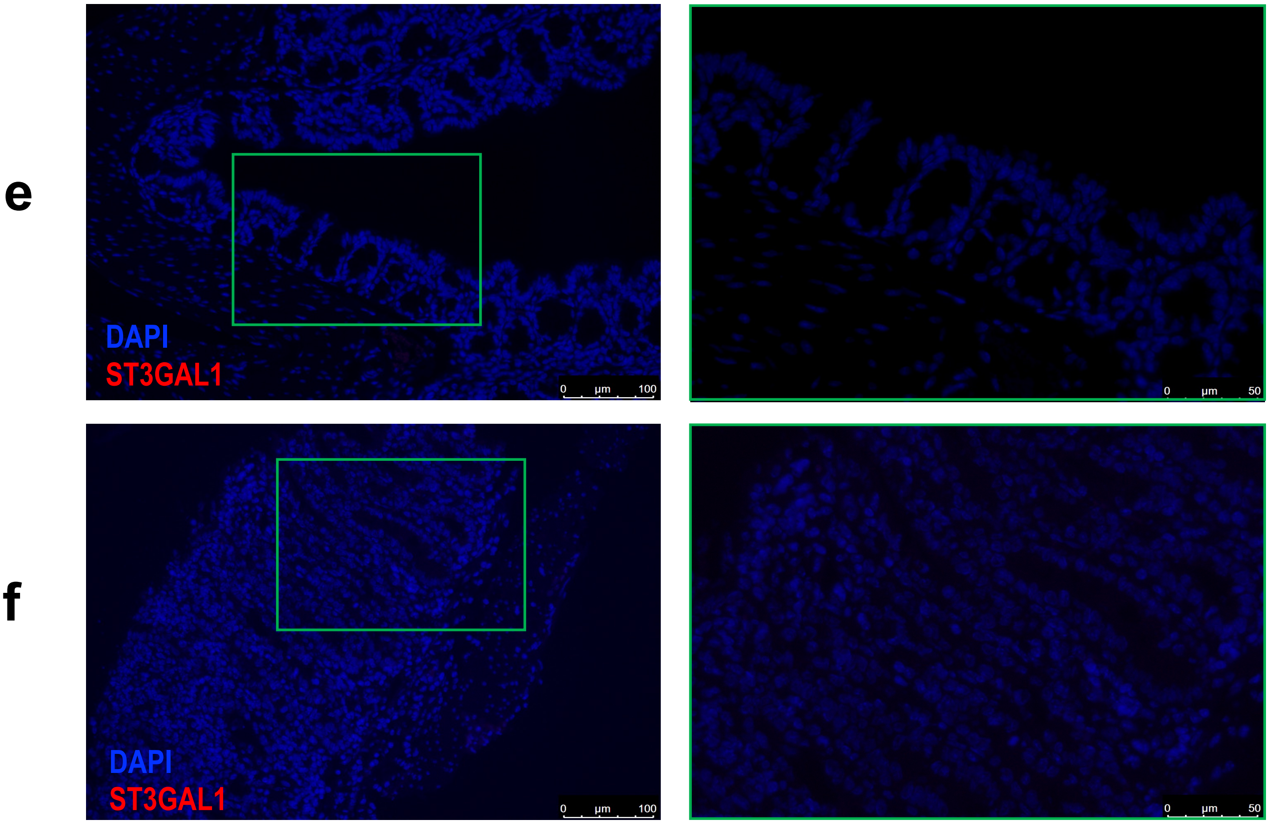

Supplement: Supplementary file 5 — Additional file 5. Detection of immunofluorescently stained ST3GAL1 in secondary abiotic IL-10−/−mice upon C. jejuni 81–176 infection. ST3GAL1 is shown in red and nuclei were stained blue using DAPI. (a + b) Immunofluorescent staining of ST3GAL1 in two individual experiments of naïve mouse colon sections. (c + d) Two individual C. jejuni infected mouse colon sections immunostained with ST3GAL1. (e) Negative controls of naïve colonic tissue sections. (f) Negative controls of colon tissue sections infected with C. jejuni. Scale bars indicate 100 µm and 50 µm (magnification). [file 13099_2023_577_MOESM5_ESM.pdf]
